# Are the Notions of Leader–Member Exchange and Organisational Citizenship Effective in Enhancing Teachers’ Job Performance in Türkiye? A Moderated Mediation Model

**DOI:** 10.3390/bs15010038

**Published:** 2025-01-02

**Authors:** Mehmet Sabir Çevik

**Affiliations:** Department of Physical Education and Sports, Siirt University, 56100 Siirt, Türkiye; mehmetsabir.cevik@siirt.edu.tr

**Keywords:** leader–member exchange, organisational citizenship, teachers’ job performance, moderating effect, moderated mediation role

## Abstract

This study is correlational and cross-sectional quantitative research that examines the moderating role of working time with the school principal on the effect of leader–member exchange on teachers’ job performance through organisational citizenship. Data were collected from 481 teachers in 43 public schools across the western, central, northern, southern, and eastern regions of Türkiye using the convenience sampling method. The Leader–Member Exchange Scale, Organisational Citizenship Scale, and Employee Performance Scale were used as data collection tools. Data were analysed through descriptive analyses, Pearson correlation analysis, and moderator and moderated mediation analyses. The results indicated positive and significant relationships among leader–member exchange, organisational citizenship behaviours, and teachers’ job performance. It was found that leader–member exchange indirectly affected teachers’ job performance through organisational citizenship, and working time with the school principal served as a moderator in the relationship between leader–member exchange and organisational citizenship. The indirect effect of leader–member exchange on job performance through organisational citizenship was stronger for teachers with shorter working durations with the principal.

## 1. Introduction

Although there is a general tendency for leaders in organisations to interact with their employees, the exchange style is emphasised to differ for each employee potentially (Graen & Uhl-Bien, 1995). The different exchanges of the organisational leader with each employee shape the attitudes and behaviours of the employees towards the organisation (Ilies et al., 2007). The leader–member exchange approach, which focuses on the exchange of the leader with their employees, draws attention to the leader’s interactional role in employees’ positive or negative behaviour towards the organisation (Breevaart et al., 2015). Similarly, there is a strong link between leader–member exchange and organisational outcomes (Borgatti & Foster, 2003), and the interaction between leaders and employees is stated to make significant and noteworthy contributions to individual and organisational outcomes (Kong et al., 2019). In other words, leader–member exchange affects the behaviours of the employees of the organisation and provides an understanding of the effects of leaders on employees (Erdoğan & Bauer, 2014).

In organisations with a high level of leader–member exchange, employees have a sense of loyalty to the organisation, exhibit constructive behaviours (Bhal et al., 2009), have increased job satisfaction (Greenberg, 2011), take on extra tasks and responsibilities for the organisation (Northouse, 2007), work harder and become motivated (Martin et al., 2016), deal with uncertain situations more, and have a heightened satisfaction with their organisation (Bauer & Green, 1996). On the other hand, in organisations where leader–member exchange is absent or low, employees’ trust in their organisation declines (Maslyn & Uhl-Bien, 2001), and they are more likely to leave their job and show cynical behaviours towards the organisation (Bommer et al., 2005). Therefore, poor interaction between managers and employees may lead to undesirable organisational behaviours (Chernyak-Hai & Tziner, 2014). Also, in the context of educational organisations, it is stated that a high level of leader–member exchange creates a trust-based environment, increases information exchange, and enables teachers to make extra effort in school-related activities (Hardianto & Sari, 2021).

How managers’ age and leadership styles impact leader–member exchange, organisational citizenship, and job performance is a significant research topic and has been broadly tackled in the literature. Studies show that such factors as age and experience mould leader–member exchange and that this exchange plays a determining role in job performance (Marks & Printy, 2003). On the other hand, a leader with more advanced age may generally lead to them adopting a more authoritarian or experiential leadership style, which may affect the quality of relationships with employees (Kark & Shamir, 2013). In fact, the impact of leadership styles on organisational citizenship behaviours is also a significant area of research. Transformational leaders may cause their employees to exert extra effort and enhance organisational commitment. This encourages organisational citizenship behaviours (Bass & Avolio, 1994; P. M. Podsakoff et al., 2000; N. P. Podsakoff et al., 2009). Therefore, a study investigating how the interaction between managers’ leadership styles and ages affects these relationships may greatly contribute to the field of organisational behaviour.

The latest research demonstrated that the high-quality interaction between leaders and members enhanced organisational citizenship and positively impacted employee job performance (Martin et al., 2016; Matta et al., 2015; Rockstuhl et al., 2012). However, a limited number of studies exist on how these interactions change depending on contextual variables. For instance, it is suggested that such situational factors as the length of the relationship may affect the impact of leader–member exchange on organisational citizenship (Dulebohn et al., 2012; He & Brown, 2013). For this reason, it is important to broaden the current literature on how the time factors in leader–member relationships affect trust, mutual support, and sense-making processes. On the other hand, one of the unique contributions of this study to the literature is the evaluation of the regulatory role of such contextual variables as the period of working with the leader. In fact, the research demonstrates that long periods of working with leaders may reinforce leader–member interactions and that these interactions may enhance their indirect effects on organisational citizenship and job performance (Erdoğan & Bauer, 2014; Thomas et al., 2019). Specifically, research on education presents a significant framework for understanding the impact of leadership processes on organisational citizenship and teachers’ job performance (Ng & Feldman, 2008). Therefore, by introducing a broader model suggestion for leadership research on individual and organisational levels, this study enables an in-depth understanding of how leader–member interaction engages with contextual factors.

Leader–member exchanges occurring specifically in educational organisations provide a flow of communication between teachers and administrators and pave the way for teachers to give healthy and meaningful feedback to their administrators (Usadolo et al., 2019). As a matter of fact, in the literature, leader–member exchange established in schools has been shown to enhance teachers’ participation in decisions (Gürler & Şimşek, 2018), leading them to exhibit positive behaviours towards the school (Turgut et al., 2015), experience less stress and burnout (Alev & Taş, 2020), make more effort for school success (Edgerson et al., 2006), feel trust and commitment to the school (Çekmecelioğlu & Ülker, 2014), and internalise innovations in the school (Zeinabadi, 2014). Some studies indicate that leader–member exchange occurring in schools enhances teachers’ organisational citizenship behaviours (P. M. Podsakoff et al., 2000; N. P. Podsakoff et al., 2009; Tekin, 2018a, 2018b) and teachers’ job performance (Turgut et al., 2015; Tekin, 2018a). However, as a result of the literature review, only one study that examines the relationships between leader–member exchange, organisational citizenship, and employee performance in educational organisations (Tekin, 2018a) and health organisations (Che et al., 2021) was found. Even though these studies (Che et al., 2021; Tekin, 2018a) examine the relationship between the three variables unitedly, they do not provide findings on how the duration of working with organisational managers affects the relationship between the variables. In other words, the present study is estimated to fill a critical gap and contribute to the literature since it aims to determine whether the relationship between leader–member exchange, organisational citizenship, and teacher performance changes regarding the duration of working with the school principal. On the other hand, determining the variables that increase teachers’ job performance can provide insights for policymakers and practitioners in countries with centralised and bureaucratic education systems such as Türkiye. In this context, the present study aims to determine whether the effect of leader–member exchange on teachers’ job performance through organisational citizenship varies according to the duration of working time with the school principal. In line with the fundamental purpose of this study, answers were sought for the below-mentioned questions:

1. Does organisational citizenship mediate the relationship between leader–member exchange and teachers’ school job performance?
2. Does the effect of leader–member exchange on organisational citizenship vary according to the period teachers work with the school principal?
3. Does the indirect effect of leader–member exchange on teachers’ job performance through organisational citizenship vary according to the period teachers work with the school principal?

### 1.1. Theoretical Framework

#### 1.1.1. Leader–Member Exchange

Leader–member exchange has recently become one of the focus topics of researchers due to the characteristics of the relationship and interaction between organisational leaders and employees (Cheung & Wu, 2012). According to leader–member exchange, which emphasises mutual relationships, the impact of the leader on the organisation and employees is shaped by the quality of the relationship established between the leader and employees (Erdoğan & Bauer, 2014). Therefore, leader–member exchange theorists point out that organisational leaders do not interact with all employees at the same level and that employees who interact with the leader tend to perform more for the organisation (Liden & Maslyn, 1998).

Leading researchers of leader–member exchange believe that leader–member exchange is multidimensional (Graen & Uhl-Bien, 1995; Liden & Maslyn, 1998). Liden and Maslyn (1998) analysed leader–member exchange in terms of emotion (influence), contribution, loyalty, and professional respect. The emotion (impact) sub-dimension of leader–member exchange refers to the sharing of emotions between the leader and the employees based on the relationship (Ahmadi et al., 2014). The contribution sub-dimension is employees’ extra work, duties, and responsibilities beyond their job descriptions to achieve organisational goals (Sullivan et al., 2003). The sub-dimension of loyalty is the leader’s and employees’ support and defence of each other under all circumstances (Liden & Maslyn, 1998). Professional respect, the last sub-dimension of leader–member exchange, is the degree of respectability of the leader and the employee within or outside the organisation (Uhl-Bien et al., 2000).

#### 1.1.2. Organisational Citizenship

Organisational citizenship behaviour (OCB) is defined as employees voluntarily contributing to the organisation’s functioning aside from their official duties and making efforts outside their roles for organisational goals (DiPaola & Tschannen-Moran, 2001). The most common classification of OCB in the literature is (Organ, 1988) classification, consisting of “altruism (cooperation/truism), courtesy, conscientiousness, sportsmanship (chivalry), and civic virtue”. In organisations, altruism (cooperation/truism) can be manifested as helping new recruits or employees with high workloads, supplying materials needed by employees, sharing work-related information with colleagues, and giving information about how to use a material (P. M. Podsakoff et al., 2000; N. P. Podsakoff et al., 2009). Courtesy is the interaction or communication of organisation employees with other organisation members (Deluga, 1995). Conscientiousness is the behaviour of employees towards the whole organisation for the organisation’s benefit (Barksdale & Werner, 2001). Sportsmanship, another dimension of OCB, means that the employees avoid all kinds of behaviours against the organisation and have positive thoughts towards it despite various negativities occurring in it (Somech & Ron, 2007). Civic virtue, the last dimension of OCB, is explained as employees’ active and responsible participation in the organisation’s management, giving importance to organisational activities and meetings, prioritising the organisation’s interests, and being interested in organisational policies (Jurewicz, 2004).

#### 1.1.3. Job Performance

Job performance refers to fulfilling a job per its purpose in terms of time, quantity, and quality (Saraswati, 2019). In the organisational context, performance is the behaviours exhibited towards organisational goals (Muthuveloo et al., 2017). Performance in organisational life is also accepted as one of the vital building blocks of human resources and organisational psychology (Aboazoum et al., 2015). Borman and Motowidlo (1993) consider job performance in two dimensions: “task performance and contextual performance”. Task and contextual performances correspond to specific behaviours of organisational employees (Griffin et al., 2000). However, task performance is a behavioural pattern that indirectly contributes to technical processes and includes organisational activities. Task performance contributes to the technical basis of the work in the organisation or emphasises the formal dimension of the work (Rotundo & Sackett, 2002). On the other hand, contextual performance is optional and voluntary behaviour outside the official task activities of the organisation (Borman, 2004). Therefore, the behaviours employees exhibit in organisations and their affective characteristics are closely related to contextual performance (Bess, 2001).

#### 1.1.4. The Mediating Effect of Organisational Citizenship in the Relationship Between Leader–Member Exchange and Teachers’ Job Performance

Recent studies have broadened the understanding of leader–member exchange and organisational citizenship. For instance, Scandura and Meuser (2022) stated the limitations of leader–member exchange as a leadership theory by reviewing the literature, adopting a questioning and critical viewpoint and claiming that returning to the role theory is necessary. Andersen et al. (2020) suggested a two-dimensional approach to leader–member exchange and stated the difference between economic and social changes. Atatsi et al. (2019) created a synthesis of the literature that tackles the issues of organisational citizenship behaviour, leader–member exchange, learning, and innovative job behaviour and concluded that it positively correlates with the performance of these elements operating under various contexts. Sidin et al. (2020) investigated the role of cultural values in the leader–member exchange and organisational citizenship behaviour among Bugis nurses and stated the importance of the cultural context. Collectively, these studies manifest the evolving nature of the research on leader–member exchange and organisational behaviour and emphasise the need for culturally sensitive approaches and theoretical improvement to understand their effect on organisational outcomes better.

The interaction between the leader and the employees increases job performance by shaping the behaviours and attitudes of the employees towards the organisation (Farrell & Oczkowski, 2012). In the literature, it is also accepted that leader–member exchange is one of the basic structures of positive organisational behaviours such as organisational citizenship (Rastgar et al., 2012). Indeed, research proves that leader–member exchange is related to organisational citizenship behaviours (Sun et al., 2013; Tekin, 2018b). This is because organisational citizenship positively affects job performance by eliminating organisational conflicts (Organ, 1988). Therefore, one of the critical outcomes that organisational citizenship is effective is job performance (Zhao & Zhou, 2019). Studies emphasise that the extra efforts of employees while performing their duties enhance the organisation’s performance significantly (Sahafi et al., 2011). Apart from these studies, others in the literature draw attention to the relationship between leader–member exchange, organisational citizenship, and job performance (Che et al., 2021; Tekin, 2018a). As a result, the following hypothesis was developed in line with the research findings in the literature.

**H1.**  *Organisational citizenship has a mediating role in the relationship between leader–member exchange and job performance.*

#### 1.1.5. Moderator Role of Working Time with the School Principal

In the emergence of organisational citizenship behaviours, demographic characteristics, in addition to the attitudes of organisational employees, can be effective (P. M. Podsakoff et al., 2000; N. P. Podsakoff et al., 2009). Similarly, it is pointed out in the literature that demographic characteristics of employees, such as education level, gender, age, and tenure, influence organisational citizenship behaviour (Kidder & McLean Parks, 1993). In this context, Rego et al. (2010) reported that gender is negatively related to sportsmanship, and men have lower scores, that age is negatively associated with sportsmanship and positively related to conscientiousness, and that tenure is negatively related to sportsmanship and positively related to conscientiousness and civic virtue. A meta-analysis study conducted by (Ng & Feldman, 2008) proved that older employees tend to exhibit more organisational citizenship behaviours than younger employees. For instance, there is a series of studies in the literature that show a correlation between employees’ job performance and education level (Lev & Koslowsky, 2012), marital status (Gede & Lawanson, 2011), gender (Inayatullah & Jehangir, 2012) and professional seniority (Sarani & Rezaee, 2017). All these studies on demographic variables that may be effective on organisational citizenship and job performance indicate that both organisational citizenship behaviours and job performance may differ according to the demographic characteristics of employees. For this reason, the following hypotheses were developed based on the fact that the duration of working time with the school principal may have a moderating effect between the research variables:

**H2.**  *The effect of leader–member exchange on organisational citizenship is moderated by the period teachers work with the school principal.*

**H3.**  *The indirect effect of leader–member exchange on teachers’ performance through organisational citizenship is moderated by the period teachers work with the school principal.*

## 2. Materials and Methods

### 2.1. Research Model

**Figure 1:**
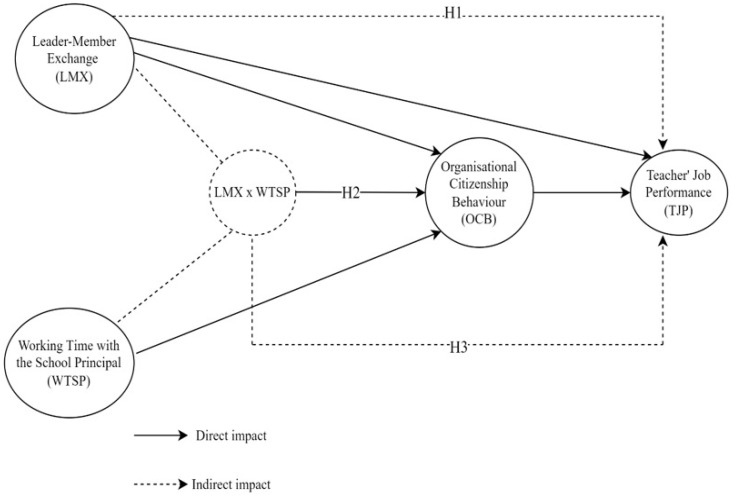
Hypothetical model of the research.

This study was designed as a correlational and cross-sectional quantitative study since it aimed to determine the moderating role of working time with the school principal in the relationship between leader–member exchange, organisational citizenship, and teachers’ job performance. In addition, the moderating effect of working time with the school principal on the relationship between leader–member exchange and teachers’ job performance through the mediating role of organisational citizenship was also tested in this study. Social scientists combine moderating and mediating effects to understand the complex nature of the relationships between variables and to obtain reliable findings (Klein & Moosbrugger, 2000). In this direction, leader–member exchange was analysed as a predictor variable, organisational citizenship as a mediator variable, teachers’ job performance as a predicted variable, and working time with the school principal as a moderator variable. The hypothetical model to be tested in the research is given in Figure 1.

### 2.2. Sampling and Data Collection Process

The study data were collected from 481 teachers working in 43 public primary, secondary, and high schools in the western, central, northern, southern, and eastern regions of Türkiye per the convenience sampling method. Convenience sampling is a recommended method, especially if there are such constraints as time, cost, and access (Taherdoost, 2016). Etikan et al. (2015) state that this method is considered preferable due to its fast and low-cost data collection advantages. This study’s focal point is solely the public schools because centralised and bureaucratic school structures are more significant in the public education system since understanding the operation of the leadership and interaction processes in the public education system in this structure may fill a substantial gap in the literature. Although random sampling could not be used in this study due to limitations such as financial burden and accessibility, importance was given to diversifying the sample in terms of school type and geographical region. Similarly, care was taken to ensure that the provinces included in this study represented the regions’ sociocultural characteristics. In this context, provinces where this study was implemented were selected to reflect the geographical regions and socio-economic differences of Türkiye. During this process, factors such as levels of regional development, population density, levels of education, and number of teachers were considered. In addition, classification methods were used to enhance the representability of provinces during the selection process, and both metropolitan and rural provinces were included in this study. This enabled this study to increase generalisability and make valid inferences in a variety of contexts.

The research data could be collected through online Google Forms via email or WhatsApp at the end of an approximately two-month period. However, a total of 23 data entries that gave the same answer to all items of the research scales were extreme values (outliers), did not show any difference, and did not show distribution within the appropriate Mahalanobis distance limits were deleted, and analyses were carried out with the data obtained from 481 teachers. The sample group consisted of 143 female (29.7%) and 338 male (70.3%) teachers; 343 (71.3%) were married, 138 (28.7%) were single; 454 (94.4%) were undergraduate graduates, 27 (5.6%) were postgraduate graduates; 183 (38%) were working in primary schools, 168 (35%) in secondary schools, and 130 (27%) in high schools. The average working time of the teachers with the school principal is 3.74 years (SD = 1.79).

### 2.3. Variables and Data Collection Tools

#### 2.3.1. Independent (Predictor) Variable: Leader–Member Exchange Scale (LMXS)

Developed by Liden and Maslyn (1998) the LMXS was adapted into Turkish by (Öztürk, 2015). Scale feeling (3 items/Sample item: I like my school principal very much as a person), contribution (3 items/Sample item: If other employees criticise me, my school principal defends me against them), loyalty (3 items/Sample item: I do not avoid doing the most challenging jobs for my school principal) and professional respect (3 items/I admire my school principal’s knowledge of the job), totalling 4 dimensions and 12 items. The LMXS is a 5-point Likert-type scale (1 = Strongly disagree, 5 = Strongly agree). CFA was conducted again on the research data to determine the construct validity of the current research. As a result of CFA, it was determined that the four-dimensional factor structure of the scale was within the appropriate ranges (RMSEA = 0.055, x2/sd = 2.447, SRMR = 0.0458, NFI = 0.97, CFI = 0.98, RFI = 0.96, AGFI = 0.94, GFI = 0.96, TLI = 0.97) (Hu & Bentler, 1995). In addition, the scale’s Cronbach’s Alpha reliability values were found to be 0.86, 0.92, 0.80, 0.83 and 0.93 for the dimensions of emotion, contribution, loyalty, professional respect and the whole scale, respectively. The Composite Reliability (CR) combined reliability values of the scale were found to be 0.87, 0.92, 0.79, 0.83 and 0.96 for the dimensions of emotion, contribution, loyalty, professional respect and the whole scale, respectively. In addition, the average variance extracted (AVE) value of the LMXS was calculated as 0.71, 0.81, 0.57, 0.62 and 0.68 in the dimensions of emotion, contribution, loyalty, professional respect, and the whole scale, respectively.

#### 2.3.2. Mediating Variable: Organisational Citizenship Scale (OCS)

This scale was developed by (DiPaola & Tarter, 2005). The Turkish version of the OCS, adapted into Turkish by Taştan and Yılmaz (2008), consists of a single dimension and 12 items (Sample items: Teachers help students in their personal time/Teachers volunteer to support extra-curricular activities). In addition, the OCS is a 5-point Likert-type scale (1 = Strongly disagree, 5 = Strongly agree). Exploratory factor analysis was performed to reveal the scale’s factor structure, and it was determined that the scale was gathered under one dimension as in the original. For this study, the construct validity and reliability of the scale were rechecked. As a result of DFA for construct validity, it was found that the research data confirmed the one-factor structure and were within the appropriate ranges (RMSEA = 0.071, x2/sd = 3.410, SRMR = 0.0303, NFI = 0.96, CFI = 0.97, RFI = 0.95, AGFI = 0.90, GFI = 0.93, TLI = 0.96) (Hu & Bentler, 1995). On the other hand, Cronbach’s Alpha reliability value of the OCS was calculated as 0.94, and the Composite Reliability (CR) was calculated as 0.95. In addition, it was determined that the AVE value of the OCS was 0.61.

#### 2.3.3. Dependent (Predicted) Variable: Employee Performance Scale (EPS)

The validity and reliability analyses of the short form of the EPS, which was originally developed by (Kirkman & Rosen, 1999), consisting of 4 items, were conducted by Sigler and Pearson (2000) in the following processes. The EPS is a 5-point Likert-type scale that is graded between “Strongly disagree (1) and Strongly agree (5)”. Sigler and Pearson (2000) calculated the Cronbach’s Alpha reliability value of the 4-item short form of EPS as 0.70. 4 items of EPS (Sample items: I complete my tasks on time/I produce solutions fastest when a problem arises) short form was adapted into Turkish by Çöl (2008). The validity and reliability analyses of the current study were redone for the EPS. It was found that the fit values related to the DFA results within the scope of construct validity of EPS were within the appropriate ranges (RMSEA = 0.000, x2/sd = 0.028, SRMR = 0.0008, NFI = 1, CFI = 1, RFI = 1, AGFI = 1, GFI = 1, TLI = 1) (Hu & Bentler, 1995). In addition, the Cronbach’s Alpha reliability value and Composite Reliability (CR) combined reliability value of EPS were found to be 0.90. On the other hand, it was found that the AVE value of the EPS was 0.70.

As can be observed, Cronbach’s alpha and combined reliability (CR) coefficients exceeding 0.70, CR values being greater than AVE (CR > AVE) and AVE being above 0.50 (Fornell & Larcker, 1981; Hair et al., 2009) and CFA fit indices being within acceptable ranges (Hu & Bentler, 1995) mean that all scales used in this research are statistically reliable and valid measurement tools at a sufficient level.

#### 2.3.4. Moderator Variable: Working Time with the School Principal

In this study, teachers’ working time with the school principal was analysed as a continuous and moderator variable. The average working time of the teachers with the school principal in years was calculated as 3.74 (SD = 1.79).

### 2.4. Data Analysis

The average working time of the teachers with the school principal in years was calculated as 3.74 (SD = 1.79). No incomplete (missing) data were found in this direction since the research data were collected online. Z scores and Mahalanobis distances were considered when determining the research’s extreme (outlier) values. Of the nine research data whose Z score was not in the range of −3 and +3 and whose Mahalanobis distance was not distributed within the appropriate limits, 14 research data that gave the same answer to all items of the scales and did not differ were deleted. The analysis assumptions were examined with the remaining 481 research data. The research data were also analysed in terms of normality distribution, linear relationship, multicollinearity problem and autocorrelation. The kurtosis and skewness values of all scales used in this study were found to be between −1.5 and +1.5. This result means the data are normally distributed (Tabachnick & Fidell, 2013). This study checked the linear relationship with the Scatter plot graph and found a linear relationship between the variables. On the other hand, VIF (variance inflation value) being in the range of 1.631 to 3.072 and less than 10; tolerance value being in the range of 0.326 to 0.613 and greater than 0.20; CI (condition index) being in the range of 10.196 to 18.316 and less than 30; and correlation values between variables being less than 0.90 (see Table 1) can be considered an indicator of a multicollinearity problem; the Durbin-Watson value being 1.969 and showing variability between 1.5 and 2.5 can be considered as an indicator that there is no autocorrelation in the research (DeMaris, 2004).

The fact that the research data were cross-sectional and collected only from teachers may cause common method bias. This study applied Harman’s one-factor test to reduce common method bias (Harman, 1967). In the present study, factor analysis with varimax rotation was performed with 28 scale items belonging to the variables, and it was observed that the results were not collected under a single factor. The factors gathered under four factors had eigenvalues above 1.0 and explained 67.904% of the total variance. In addition, in order to reduce social desirability and method bias in the research, the scales were presented clearly and understandably in the order of dependent, mediator, moderator, and independent variables and the principle of anonymity was followed throughout the research (MacKenzie & Podsakoff, 2012).

The analyses of the research were carried out according to the determined hypotheses. In this context, descriptive and Pearson product–moment correlation analyses of this study were conducted with SPSS 26; mediated SEM (Structural Equation Model) analysis with latent variable (H1) was conducted with AMOS 24; moderator analysis (H2) and moderated mediation analysis (H3) were conducted with PROCESS Macro v4.2 plug-in. In addition, the results of the moderator (H2) and moderated mediation (H3) analyses were tested with ‘Model 1’ and ‘Model 8’ from PROCESS Macro models, respectively. All statistical values in this study were determined using a 95% Confidence Interval and a 5000 bootstrap sample method. The bootstrap method evaluated statistical significance using a confidence interval that does not contain zero. Additionally, in the research, short- and long-term study definitions were made operational by determining a standard cut-off point while conducting a moderation analysis through PROCESS macro. To do this, in order to more consistently analyse the effect of the working length variable, lower and upper quartile values were taken as references based on the distribution in the sampling (Hayes, 2013).

## 3. Results

### 3.1. Findings Related to Descriptive Statistics and Correlation Analysis

The results of descriptive statistics and correlation analysis for leader–member exchange, organisational citizenship, and teachers’ job performance are shown in Table 1.

As can be seen from Table 1, the mean of all variables ranges between 3.22 and 3.55. In terms of the total scores of the scales, the order from the lowest mean to the highest mean is as follows: organisational citizenship (M = 3.36, SD = 0.83), leader–member exchange (M = 3.43, SD = 0.89), and employee performance (M = 3.50, SD = 0.99). On the other hand, it was found that the standard deviation values of the research scales ranged between 0.83 and 1.05 and that the teachers had the most similar thoughts about organisational citizenship (SD = 0.83), and the variable they differed the most was the contribution dimension of leader–member exchange (SD = 1.05). According to the results of the correlation analysis of this study, there were significant positive relationships between all variables. When the relationships between the variables were examined, it was found that there were significant positive relationships between leader–member exchange and organisational citizenship (r = 0.61, *p* < 0.01), employee performance (r = 0.48, *p* < 0.01), and between organisational citizenship and employee performance (r = 0.60, *p* < 0.01).

### 3.2. Findings Related to Mediation Analysis of the Research

**Figure 2:**
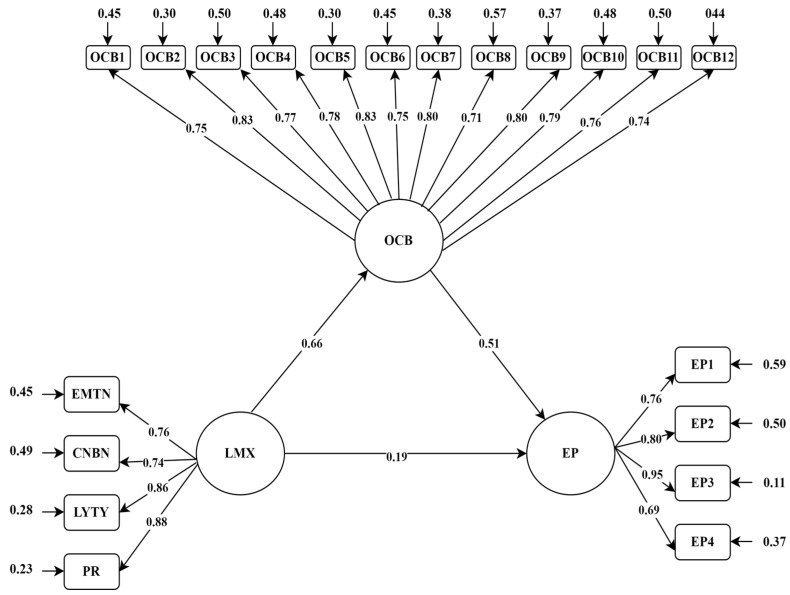
SEM analysis diagram of this study with latent variable.

In order to test whether organisational citizenship has a mediating role in the relationship between leader–member exchange and job performance, an (indirect-mediation) SEM analysis with latent variables mediation was conducted. Accordingly, leader–member exchange was analysed as the predictor variable, organisational citizenship as the mediator variable, and teachers’ job performance as the predicted variable. Figure 2 shows the latent variable SEM analysis diagram for the prediction of teachers’ performances (EMTN: “emotion“ dimension of the leader–member exchange scale; CNBN: “contribution“ dimension of the leader–member exchange scale; LYTY: “loyalty“ dimension of the leader–member exchange scale; PR: “Professional respect“ dimension of leader–member exchange scale; LMX; Leader–Member Exchange Scale; OCB: Organisational Citizenship Behaviour Scale; OCB1...OCB12: Items of the Organisational Citizenship Behaviours Scale; EP: Employee Performance Scale; EP1...EP4: Items of Employee Performance Scale).

As seen in Figure 2, the fit values for the latent variable SEM analysis were found to be within statistically appropriate ranges (RMSEA = 0.070, x2/sd = 3.36, SRMR = 0.0408, NFI = 0.92, CFI = 0.95, RFI = 0.91, AGFI = 0.86, GFI = 0.89, TLI = 0.94) (Hu & Bentler, 1995). However, according to the results of SEM analysis with latent variables, it was found that leader–member exchange significantly and positively predicted organisational citizenship behaviours (β = 0.66, SE = 0.043, t = 13.239, 95% CI [0.590, 0.719]) and teacher performance (β = 0.19, SE = 0.057, t = 3.402, 95% CI [0.079, 0.307]); and organisational citizenship behaviour significantly and positively predicted teacher performance (β = 0.51, SE = 0.071, t = 8.446, 95% CI [0.395, 0.617]). Accordingly, leader–member exchange predicts organisational citizenship behaviours, organisational citizenship behaviours predict employee performance at a large effect level, and leader–member exchange predicts employee performance at a small effect level (Kline, 2013).

The results of the 5000 bootstrapping analyses conducted to determine the statistical significance of the indirect effect of leader–member exchange on teacher performance through organisational citizenship are given in Table 2.

As seen in Table 2, the indirect effect of leader–member exchange on teachers’ performance through organisational citizenship was found to be significant and positive (Indirect Effect = 0.34; *p* = 0.000, 95% CI [0.257, 0.414]). In other words, the difference between the total and direct predictive power of leader–member exchange on teachers’ performance is statistically significant. The total impact value of the research is large, the direct impact value is small, and the indirect impact value is medium (Kline, 2013). In this context, since the indirect effect results of the research are significant, the H1 hypothesis is accepted and confirmed.

### 3.3. Findings Related to the Moderator Role of Working Time with the School Principal

The results of the regression analysis conducted to test the hypothesis “H2: The effect of leader–member exchange on organisational citizenship is regulated by the length of time teachers work with the school principal” are presented in Table 3. The results of regression analysis on the moderating effect of teachers’ working time with the school principal on the effect of leader–member exchange on organisational citizenship.

As can be observed in Table 3, according to the results of the analyses related to the moderating effect, leader–member exchange (b = 0.7755, t = 10.0565, 95% Confidence Interval [0.6239, 0.9270], *p* < 0.01) and working time with the school principal (b = 0.1771, t = 2.7195, 95% Confidence Interval [0.0492, 0.3051], *p* < 0.01) had a significant positive effect on organisational citizenship. It was determined that the interactional effect of the moderator variable (Leader–Member Exchange X Working Time with the School Principal) on organisational citizenship had a significant impact (b = −0.0539, t = −2.8838, 95% Confidence Interval [−0.0906, −0.0172], *p* < 0.01) and all variables included in this study explained 38.95% of the change in organisational citizenship. Moreover, the additional variance explained by the exchange variable was found to be 1.06%. These results indicate that the length of time teachers work with the school principal plays a moderating role in the relationship between leader–member exchange and organisational citizenship, and the H2 hypothesis of this study is accepted. The statistical representation of the model for the moderator effect is as follows (X: Leader–Member Exchange, Y: Organisational Citizenship, W: Period of working with the school principal, XW: Exchange Variable):

Y = 0.7306 + 0.7755X + 0.1771W − 0.0539XW

**Figure 3:**
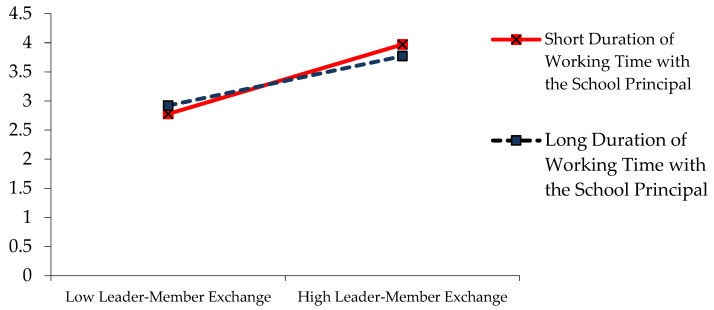
Simple slope graph according to the duration of working time with the school principal.

In order to better understand the role of the duration of working time with the school principal in the relationship between leader–member exchange and organisational citizenship, a simple slope graph was drawn (see Figure 3).

As shown in Figure 3, the leader–member exchange is located on the *X*-axis of the simple slope graph, and the level of teachers’ organisational citizenship behaviours is on the *Y*-axis. The duration of working time with the school principal was analysed as a moderator variable in the relationship between leader–member exchange and organisational citizenship. In this framework, the relationship between leader–member exchange and teachers’ organisational citizenship levels was found to show a change in a positive direction and significantly based on short duration (b = 0.7216, t = 11.8647, 95% Confidence Interval [0.6021, 0.8411], *p* < 0.01), medium duration (b = 0.5600, t = 16.5479, 95% Confidence Interval [0.4935, 0.6265], *p* < 0.01), and long duration (b = 0.4522, t = 8.3512, 95% Confidence Interval [0.3458, 0.5586], *p* < 0.01) of working time with the school principal. In other words, the effect of leader–member exchange on organisational citizenship is higher when the working time with the school principal is short, medium, and long. However, the effect of leader–member exchange on organisational citizenship is stronger when working time with the school principal is short than when working time with the school principal is medium and long.

### 3.4. Findings Related to the Moderated Mediation Role of Working Time with the School Principal

The results of the moderated mediation analyses conducted to test the hypothesis “H3: the indirect effect of leader–member exchange on teachers’ performance through organisational citizenship is moderated by the length of time teachers work with the school principal“ are shown in Table 4.

**Figure 4:**
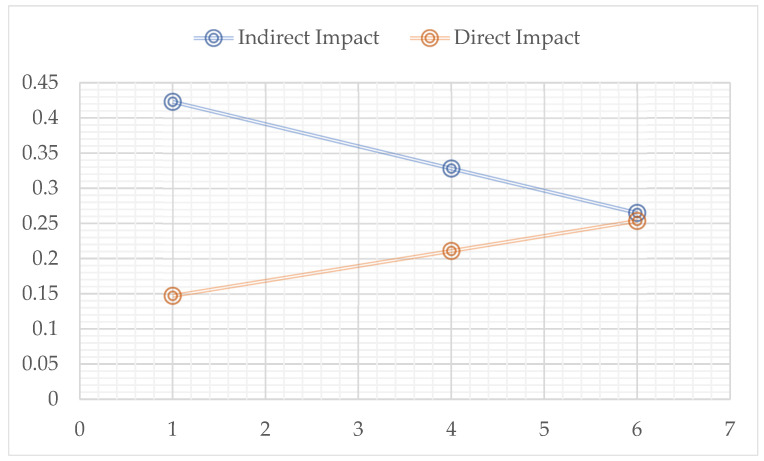
Graphical representation of the moderated mediation effect.

As seen in Table 4, it was examined whether the indirect effect of leader–member exchange on teachers’ job performance through organisational citizenship varies according to the duration of working time with the school principal. The significance of the moderated mediation index (b = −0.0316, 95% Confidence Interval [−0.0581, −0.068]) means that working time with the school principal plays a moderating role in the indirect effect of leader–member exchange on teachers’ performance through organisational citizenship. In other words, the indirect effect of leader–member exchange on teachers’ performance through organisational citizenship differs statistically significantly according to whether teachers’ duration of working time with the school principal is short (b = 0.4230, 98% Confidence Interval [0.3108, 0.5423]), medium (b = 0.3282, 98% Confidence Interval [0.2444, 0.4139]) and long (b = 0.2651, 98% Confidence Interval [0.1725, 0.3695]). In this context, the H3 hypothesis is accepted. In order to better understand the moderated mediation role of the research, a slope analysis graph was drawn (see Figure 4).

As shown in Figure 4, the effect of leader–member exchange on teachers’ performance through organisational citizenship was realised at any duration of working with the school principal. In other words, the effect of leader–member exchange on teachers’ performance through organisational citizenship differs significantly when the duration of working with the school principal is short, medium, and long. It was found that the indirect effect of leader–member exchange on teachers’ performance through organisational citizenship was stronger for teachers with a short duration of working with the school principal than for teachers with a medium and long duration of working with the school principal.

## 4. Conclusions and Discussion

One of the critical results of this study was that organisational citizenship played a mediating role in the relationship between leader–member exchange and teacher performance in schools. It is known that leader–member exchange increases employees’ organisational citizenship behaviours (Sun et al., 2013) and that organisational citizenship behaviours affect employee performance (Organ, 1988). In one aspect, it is accepted that leader–member exchange is one of the main precursors of organisational citizenship (Rastgar et al., 2012). As the leader–member exchange becomes more frequent in schools, teachers will likely undertake extra tasks, and their performance will increase accordingly. As a matter of fact, some studies in the literature drawing attention to the relationship between leader–member exchange and organisational citizenship and job performance (Che et al., 2021; Tekin, 2018a) support this conclusion. The impact of leader–member exchange on organisational citizenship is also known to vary depending on cultural context (Yu et al., 2018). The relationship between leader–member exchange and organisational citizenship is stronger in the Western individualistic and low power distance contexts compared to collectivist high power distance contexts (Rockstuhl et al., 2012). Studies on cultural differences underscore that individual cultural values affecting the relationship between leader–member exchange and job results demonstrate differences in each society (Erdogan & Liden, 2006; Maslyn & Uhl-Bien, 2001). In this context, cultural value tendencies are accepted as individual characteristics that mould the effects of leadership and identify subordinates’ perceptions, attitudes, and behaviours (Hee et al., 2020; Kirkman et al., 2009). Consequently, the inferences of the cultural context of leader–member exchange are dissimilar to non-Western collectivist and high-power distance societies (Anand et al., 2018; Terpstra-Tong et al., 2020). For this reason, the current study presents significant contributions to education systems showing similar social and cultural characteristics by tackling leader–member exchange and organisational citizenship behaviour in the context of Türkiye. At the same time, the effect of leader–member exchange on teachers’ performance through organisational citizenship can be considered vital for countries like Türkiye, where centralised and bureaucratic education systems are implemented. This is because Türkiye is among the countries with high power distance that shapes social interactions (Çapan Özeren et al., 2022). Accordingly, in Türkiye, where power distance is high, school principals’ interaction with teachers may have increased teachers’ organisational citizenship behaviours, and organisational citizenship behaviours may have enhanced teachers’ job performance.

This study determined that the relationship between leader–member exchange and teachers’ organisational citizenship behaviours varied depending on the duration of working with the school principal. However, the effect of leader–member exchange on organisational citizenship is stronger when the duration of working with the school principal is short compared to when it is medium or long. Podsakoff et al. (P. M. Podsakoff et al., 2000; N. P. Podsakoff et al., 2009) states that demographic characteristics, as well as attitudes towards the organisation, are effective in the extra effort of employees in organisations. Similarly, Kidder & McLean Parks (1993) found that educational level, gender, age, and tenure in organisational citizenship behaviours; Thevi and Priya (2022) found that age, gender, and academic status; Nijhawan et al. (2022) noted that age; Monga et al. (2017) pointed out that age; educational status and tenure; and Rego et al. (2010) found that gender, age, and tenure affect organisational citizenship behaviour. On the other hand, Ng and Feldman (2008) reported in a meta-analysis study that older employees tend to exhibit more organisational citizenship behaviours than younger employees. In contrast to these studies, Deckop et al. (1999) found that age, academic level and gender and Badawy et al. (2017) found that gender, age, years of experience and education level had no significant effect on organisational citizenship. The relationship between leader–member exchange, organisational citizenship, and teacher job performance is critical for the sustainable success of educational institutions. In other words, the period of working with the principal quite likely plays a determining role in the leader–member exchange and, thus, it impacts such consequences as organisational citizenship and job performance in the long term. Short- or long-term working relations with the principle and a stronger trust between the leader and the subordinate may enhance teachers’ organisational commitments and job performances by creating communication and a commitment environment. This perspective demonstrates that our findings are not only restricted to the current context but may also have significant applications in various organisations. In this direction, this study has made a unique contribution to the literature since it has shown the moderating effect of working time with the school principal.

Another significant study result is that the effect of leader–member exchange on teachers’ job performance through organisational citizenship is moderated by the duration of working time with the school principal. Empirical studies on both organisational citizenship (Badawy et al., 2017; Kidder & McLean Parks, 1993; Ng & Feldman, 2008; Rego et al., 2010; Thevi & Priya, 2022) and employee performance (Gede & Lawanson, 2011; Lev & Koslowsky, 2012) emphasise that organisational citizenship and employee performance differ depending on demographic variables. It is very important for the present study to draw attention to the determinant of the duration of working with school principals as a different demographic variable in countries like Türkiye, which have centralised school systems with a predominantly bureaucratic and hierarchical structure. In this context, the results of this study, depending on the length of time working with the school principal, indicate that leader–member exchange can play an important role in increasing the performance of the members of the organisation (Ilies et al., 2007) and that leader–member exchange can occur through organisational citizenship behaviours (Che et al., 2021). On the other hand, organisational citizenship behaviours are known to enable employees to contribute to their institutions by going beyond their job descriptions (DiPaola & Tschannen-Moran, 2001) and to have a positive effect on teacher performance (Arifin, 2022). Therefore, in situations where leader–member exchange is high, teachers are more likely to exhibit organisational citizenship behaviours, and this is directly reflected in their performance (Cohen & Liu, 2011). However, in this study, the fact that teacher performance was stronger in teachers who had a short duration of working time with the school principal may be attributed to the support, guidance, and motivation that teachers who are relatively new to working with the school principal receive from their school principals.

## 5. Practical Implications

The present study provides evidence that leader–member exchange affects teachers’ job performance through organisational citizenship, depending on the duration of working time with the school principal. For this reason, school principals can be supported in gaining effective communication and empathy skills by considering that teachers can perform better in schools where the leader–member exchange is robust. Principal-teacher exchange can be strengthened by giving weight to teamwork and school social activities. Such activities can positively affect teachers’ overall performance by increasing their organisational citizenship behaviours. On the other hand, leader–member exchange and organisational citizenship criteria can be added to teacher performance systems. Therefore, it may be possible to measure teachers’ performance not only in academic terms but also in terms of their contribution to the school. Teachers can be more involved in decision-making processes. Teachers’ feeling that their opinions and suggestions are valued in school management can increase their organisational citizenship behaviours, and organisational citizenship behaviours can improve their job performance.

Improving the leader–member exchange organisational citizenship behaviour is critical in reinforcing the relationships of school managers with teachers and supporting the school culture. Per our findings, strategic training programmes may be recommended for managers. For instance, programmes that would improve empathic skills for school principals to better understand teachers’ needs and concerns can be organised. Active listening techniques enable teachers to feel valued. In addition, training principals on effective and motivating feedback may enhance the quality of exchange with teachers. On the other hand, school principals ought to learn to establish a school culture in which collaboration and volunteering are in the foreground to enhance teachers’ organisational citizenship behaviours. In this scope, teamwork practices and reward systems can be suggested to school principals. School teachers should be taught strategies for acknowledging teachers’ efforts and achievements. For instance, clear statements of appreciation or symbolic rewards may encourage teachers to assume more responsibilities. In addition, school principals can experience the practices of improving leader–member exchange and organisational citizenship behaviours in various school scenarios. For example, exercises can be organised using simulations built on challenging teacher-principal interactions or team clashes. Under the guidance of experienced principals, model practices on improving leader–member exchange may benefit leader–member exchange and organisational citizenship. Besides, actions teaching managers how to support and appreciate teachers, bearing in mind individual differences, may also encourage improving their organisational citizenship behaviour. In this scope, special training programmes to be used in various school environments may materialise the effect of leadership behaviours on teacher performance. To illustrate, practices on principals’ habits of regular feedback to teachers, establishing open communication channels, and providing individual support may enhance leader–member exchange. Such strategies reinforce teachers’ organisational behaviours and job performances and also contribute to establishing a more positive ambience in school environments.

## 6. Theoretical Implications

Studies exist in today’s literature on the significant impact of cultural context on leadership and organisational behaviours. In this scope, Hofstede (1980) explain how the understanding of leadership in different cultures is formed and foresees the development of a more hierarchical leadership style through high power distance. Leadership may be more authoritarian and centralised in countries with high power distances, such as Türkiye. Gelfand et al. (2007) showed how cultural norms and values shaped leadership practices and team exchanges. These findings shed light on how the leadership styles in Türkiye, and especially the interaction styles of school managers in education, may differentiate compared to countries with different cultures. House et al. (2004) discussed how leadership styles and cultural dimensions affect teachers’ job performances and organisational commitments and stated that leadership practices differ from culture to culture. These studies demonstrate that the leadership practices in different countries may diversify more compared with the findings in Türkiye and how leadership styles in education may become harmonious with cultural context.

The leader–member exchange is even more critical in centralised and bureaucratic education systems such as Türkiye. Therefore, the study results may provide new insights and perspectives on how leader–member exchange may function in different structural and cultural contexts. This study contributes to how organisational citizenship can play a mediating role in increasing teacher performance according to the duration of working time with the school principal. Therefore, the study results can direct the theoretical discussions on how working time with the school principal reflects on teachers’ job performance. On the other hand, based on the results of the current study, theoretical models can be developed on the dynamics and effects of leadership in centralised and bureaucratic education systems, how leadership evolves, and how it contributes to teacher performance.

## 7. Limitations of This Study and Suggestions for Future Research

The results of this study should be evaluated within the framework of some limitations. Although the participant group of this study consisted of teachers working in public schools in different regions of Türkiye (west, centre, north, south and east), it should be recognised that they do not represent all teachers in Türkiye. In other words, collecting data from teachers working in other regions of Türkiye and private schools may yield different results. Another limitation concerns the sampling method used in this study. Even though convenience sampling was preferred for this study due to practical limitations and challenges in data collection processes, other sampling methods, such as stratified sampling, may be adopted for the results of future studies to be more comprehensive and representative. The fact that data were collected through self-report in this study can be observed as another limitation. The teachers filled out all scales of the study. This may have led teachers to adopt a subjective attitude towards answering the scales. Teachers may have reported their desired results instead of evaluating the current situation in the research. In order to minimise this problem in future studies, school principals, in addition to teachers, can be included in the study. Another limitation of the research is related to the methodological design. This study’s cross-sectional design covers a single timeframe and cannot fully prove the causal relationships between the variables. Longitudinal or experimental studies can determine causal relationships between variables. On the other hand, since the research data were collected as a whole, it is unknown which data belong to which school. Therefore, this study could not make inferences about the school level. Analyses with multi-level models can provide a more accurate interpretation of the results. However, examining the differences with clustering analysis by forming homogenous groups on the basis of the demographic characteristics of participants is a valuable contribution that would enhance methodological depth, bearing in mind other new studies. Such an approach may allow for a clearer understanding of the leader–member exchange and employee behaviour dynamics in various sub-groups. In addition, to have a deeper understanding of the relationship among leader–member exchange, organisational citizenship, and job performance, analysing moderator variables such as cultural dimensions and leadership styles may provide significant contributions. Specifically, cultural dimensions such as power distance and individualism-collectivism may significantly shape teachers’ perceptions of leadership processes and the reflection of these perceptions on job results. In addition, analysing the effects of such different leadership styles as transformational or distributed leadership on leader–member exchange and organisational citizenship may explain how this relationship operates under various contexts.

## Figures and Tables

**Table 1:** Descriptive statistics and correlation analysis results (*n* = 481).

Variables	M	SD	LMX	EMTN	CNBN	LYTY	PR	OCB	EP
LMX	3.43	0.89	1						
EMTN	3.53	1.03	0.84 **	1					
CNBN	3.22	1.05	0.83 **	0.59 **	1				
LYTY	3.42	1.04	0.88 **	0.65 **	0.62 **	1			
PR	3.55	1.00	0.89 **	0.66 **	0.64 **	0.78 **	1		
OCB	3.36	0.83	0.61 **	0.48 **	0.55 **	0.53 **	0.55 **	1	
EP	3.50	0.99	0.48 **	0.45 **	0.37 **	0.44 **	0.40 **	0.60 **	1

** *p* < 0.01, M = Mean, SD = Standard Deviation, LMX: Leader–Member Exchange, EMTN: Emotion, CNBN: Contribution, LYTY: Loyalty, PR: Professional Respect, OCB: Organisational Citizenship Behaviour, EP: Employee Performance.

**Table 2:** Bootstrapping analysis results for indirect effect.

Impacts	*β*	SE	*p*	Bootstrapping	
				95% Confidence Interval	
				Lower Threshold	Upper Threshold
Total Impact	0.53	0.051	0.000 *	0.453	0.594
Direct Impact	0.19	0.057	0.008 *	0.079	0.307
Indirect Impact	0.34	0.049	0.000 *	0.257	0.414

* *p* < 0.01, *β*: Standardised beta coefficient, SE: Standard error.

**Table 3:** The results of regression analysis on the moderating effect of teachers’ working time with the school principal on the effect of leader–member exchange on organisational citizenship.

Variables **	*b*	SE	*p*	*t*	95% Confidence Interval	
					Lower Threshold	Upper Threshold
Constant	0.7306	0.2701	0.0071 *	2.7053	0.1999	1.2613
Leader–Member Exchange (X)	0.7755	0.0771	0.0000 *	10.0565	0.6239	0.9270
Working Time with the School Principal (W)	0.1771	0.0651	0.0068 *	2.7195	0.0492	0.3051
Exchange Variable (X * W)	−0.0539	0.0187	0.0041 *	−2.8838	−0.0906	−0.0172
R = 0.6241, R^2^ = 0.3895, Adjusted R^2^ = 0.0106						

* *p* < 0.01, *b*: unstandardised beta coefficient, SE: standard error, ** working time with the school principal was analysed as a continuous variable as “years”.

**Table 4:** Moderated mediation analysis results.

Indirect Effect **	*b*	SE	95% Confidence Interval	
			Lower Threshold	Upper Threshold
1 (Short duration of working time with the school principal)	0.4230 *	0.0589	0.3108	0.5423
4 (Medium duration of working time with the school principal)	0.3282 *	0.0428	0.2444	0.4139
6 (Long duration of working time with the school principal)	0.2651 *	0.0495	0.1725	0.3695
Moderated Mediation Index	−0.0316 *	0.0130	−0.0581	−0.0068

* *p* < 0.05, *b*: unstandardised beta coefficient, SE: standard error, ** working time with the school principal was analysed as a continuous variable as “years”.

## Data Availability

Data are available from the author upon reasonable request.
